# Chromatin looping and eRNA transcription precede the transcriptional activation of gene in the β-globin locus

**DOI:** 10.1042/BSR20140126

**Published:** 2015-03-18

**Authors:** Yea Woon Kim, Sungkung Lee, Jangmi Yun, AeRi Kim

**Affiliations:** *Department of Molecular Biology, College of Natural Sciences, Pusan National University, Busan 609-735, Korea

**Keywords:** chromatin looping, enhancer RNAs (eRNAs), mouse β-globin locus, transcriptional activation, 3C, chromosome conformation capture, eRNA, enhancer RNA, HS, hypersensitive site, LCR, locus control region, MEL, murine erythroleukaemia, RT, real-time

## Abstract

Enhancers are closely positioned with actively transcribed target genes by chromatin looping. Non-coding RNAs are often transcribed on active enhancers, referred to as eRNAs (enhancer RNAs). To explore the kinetics of enhancer–promoter looping and eRNA transcription during transcriptional activation, we induced the β-globin locus by chemical treatment and analysed cross-linking frequency between the β-globin gene and locus control region (LCR) and the amount of eRNAs transcribed on the LCR in a time course manner. The cross-linking frequency was increased after chemical induction but before the transcriptional activation of gene in the β-globin locus. Transcription of eRNAs was increased in concomitant with the increase in cross-linking frequency. These results show that chromatin looping and eRNA transcription precedes the transcriptional activation of gene. Concomitant occurrence of the two events suggests functional relationship between them.

## Abstract 2

Chromatin looping between enhancer and promoter was generated after chemical induction but before the transcriptional activation of gene in the β-globin locus. Transcription of enhancer RNAs was increased in concomitant with the increase of chromatin looping in this locus.

## INTRODUCTION

Enhancers are regulatory elements activating the transcription of target genes. They are usually located at long distance from the genes in genome. The transcriptional activation of the genes depending on cell differentiation or experimental induction brings enhancers to promoters in close proximity, generating loop structure in nuclear environment [1,2]. This conformational change suggests that chromatin looping has a positive role in the transcriptional activation of genes. The positive role is supported by transcriptional inactivation, accompanying the loss of loop structure in cells where chromatin loop mediating proteins are depleted [3–5] and by transcriptional activation in a locus having forced chromatin looping [6]. However, it is not clear when the chromatin loop structure is formed for transcriptional activation of gene and whether the looping precedes the transcriptional activation of genes.

Earlier studies report that noncoding RNAs are transcribed on enhancers [referred to as enhancer RNAs (eRNAs)] and transcription of eRNAs correlates with transcription of target genes [7–9]. These reports propose the functional mechanism of enhancer through the elongation of RNA polymerase II or eRNAs *per se*. eRNAs are transcribed in a transcription factor-dependent manner [10]. The transcription of eRNAs appears to play a role in depositing mono- and dimethylation of histone H^3^K^4^ at *de novo* enhancers by preceding these modifications [11]. A study using siRNA shows that eRNAs regulate chromatin accessibility and RNA polymerase II occupancy at the target genes [12]. Transcriptional repressors have been reported to function by inhibiting the transcription of eRNAs in distal enhancers [13].

The β-globin locus has the locus control region (LCR) at upstream region of the globin genes. The LCR consists of several DNase I hypersensitive sites (HSs) that contain binding motifs for transcription activators and functions as an enhancer to regulate the spatio-temporal transcription of the globin genes. When the globin gene is actively transcribed, the LCR HSs are positioned in close proximity with the active gene, forming a chromatin loop [14–16]. In addition, non-coding RNAs are synthesized from the LCR in erythroid cells with the association of RNA polymerase II [17–21]. The RNAs were reported to be transcribed from upstream region of LCR HS5 or from many sites of HS2 toward the downstream globin genes [21–23]. The transcription of non-coding RNA from the LCR HS2 accompanies locus wide histone acetylation between the HS2 and target globin gene in minichromosomal locus [24].

The β major globin gene of the mouse β-globin locus can be transcriptionally-induced in murine erythroleukaemia (MEL) cells by chemical treatment. Chromatin structure in the β-globin locus in uninduced MEL cells, such as hypoacetylation of histones and weak association of RNA polymerase II and transcription activators, is strongly activated by the chemical induction [3,25–27]. In our previous study, performed over a time course, transcription of the β-major-globin gene was substantially increased at 48 h after chemical induction, but not at 24 h [28]. Further increase in transcription was observed at 72 h. Sequential analysis of chromatin structure, such as transcription activator binding and covalent modifications at histone lysine residues, showed the kinetics of chromatin structural changes in the LCR and target gene during transcriptional induction and revealed the correlation of the changes with gene transcription. In the present study, to ask the kinetics of chromatin looping and eRNA transcription during transcriptional induction, we analysed the mouse β-globin locus in a time course manner using MEL cells chemically treated. The results show that these events, chromatin looping and eRNA transcription, precede the transcriptional activation of gene and take place together during transcriptional induction procedure.

## MATERIALS AND METHODS

### Cell culture

MEL cells were grown in Dulbecco's Modified Eagle's medium (DMEM) containing 10% FBS. To transcriptionally activate the β-major-globin gene, MEL cells at 1.5×10^5^/ml of density were treated with 5 mM of HMBA (10-[(3-Hydroxy-4-methoxybenzylidene)]-9(10*H*)-anthracenome) for 24, 48 and 72 h [29].

### Quantitative RT-PCR

RNA was prepared from 2×10^6^ MEL cells using the RNeasy Plus Mini Kit (Qiagen). A half microgram of RNA was reverse transcribed with random hexamers using the Superscript III first-strand synthesis system as suggested by the manufacturer (Invitrogen). A half microgram of RNA was reacted without reverse transcriptase. cDNA was amplified in a 10 μl of reaction volume by quantitative PCR using TaqMan chemistry. The relative intensity of specific cDNA sequences was compared with a genomic DNA standard using the comparative Ct method and then normalized with the relative intensity for the actin. Three independent preparations of RNA were analysed. The sequences of primers and TaqMan probes were provided in our previous study [28].

### Chromosome conformation capture

Chromosome conformation capture (3C) assay was performed, as described, with the reduced number of cells [15,30]. MEL cells were cross-linked with 1% formaldehyde and nuclei were prepared from approximately 1–2×10^6^ cells. Eight hundred units of *Hin*dIII restriction enzyme were used to digest DNA for overnight and the digested DNA was ligated with T4 ligase. The ligated DNA was purified after reverse cross-linking. The 3C products were quantitatively amplified by PCR using SYBR Green as a fluorescence dye. To correct the differences of ligation efficiency between fragments and the difference of PCR efficiency between primer sets, control templates were prepared by digesting and ligating equimolar amounts of bacterial artificial chromosome (BAC) vectors containing the β-globin locus and Ercc3 (excision repair cross-complementation group 3) gene and same amount of genomic DNA [14]. The ligation between the two fragments was analysed using the reverse direction primer of each fragment as shown in Figure 2(A). The relative cross-linking frequency between two fragments was determined by comparing DNA ligated in cross-linked 3C samples with DNA ligated randomly in control templates and then by normalizing with the cross-linking frequency in the Ercc3 gene [31]. Sequences of primers for 3C assay are presented in Supplementary Table S1.

### ChIP

ChIP was carried out as described [32]. Briefly, MEL cells (2×10^7^) were cross-linked with 1% formaldehyde and then nuclei were isolated by cell lysis. Chromatin was fragmented by MNase digestion and sonication into mainly mononucleosomes and then was incubated with RNA polymerase II antibodies (Santa Cruz Biotechnology) or normal rabbit IgG (Santa Cruz Biotechnology) after pre-clearing. Protein–DNA complexes were recovered with protein A agarose beads and DNA was purified after reverse cross-linking.

## RESULTS

### Chromatin looping between the LCR HSs and active gene precedes the transcriptional activation of gene in the mouse β-globin locus

**Figure 1:**
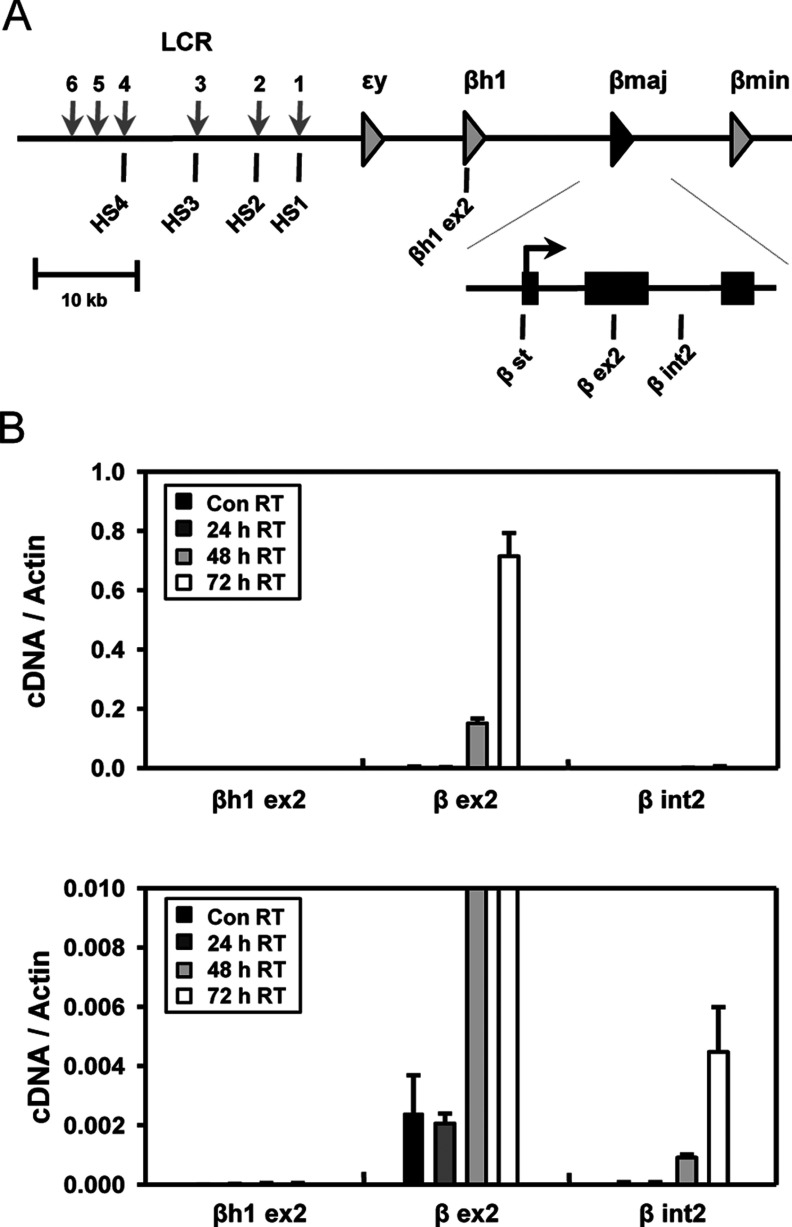
Transcript levels of the β-major-globin gene in HMBA-treated MEL cells (**A**) The mouse β-globin locus is presented. Vertical grey arrows indicate DNase I HSs in the LCR. The exons of the β-major-globin genes are represented by black squares in the extended diagram. Vertical bars named below the diagram denote the locations of TaqMan amplicons used in RT-PCR. (**B**) cDNA was prepared from RNA isolated from MEL cells at uninduced state (Con) and induced states for 24, 48 and 72 h with HMBA. Transcript levels of the βh1 and β-major-globin gene were measured at the four stages by comparing with transcript levels of the actin control gene. Same data were represented with different *y*-scale in a bottom graph. The results are averages of 3–4 independent experiments ± S.E.M.

To ask the kinetics of chromatin looping between enhancer and promoter during transcriptional induction, we induced the mouse β-globin locus in MEL cells by treating with HMBA for 3 days (Figure 1A). The β major globin gene transcripts were analysed at every 24 h by amplifying the exon 2 and intron 2 regions in quantitative real-time (RT)-PCR (Figure 1B). As expected, a basal level of transcription in the β major globin gene was not changed at 24 h after the HMBA treatment and was remarkably increased at 48 h with further increase at 72 h. This transcriptional induction pattern was confirmed by analysing the intron 2 region for preRNAs.

**Figure 2:**
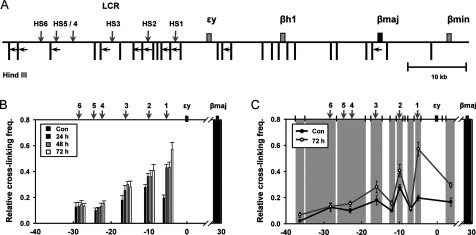
Relative proximity between the LCR HSs and β-major-globin gene in HMBA-treated MEL cells (**A**) 3C assay was performed with *Hin*dIII restriction enzyme. *Hin*dIII sites and PCR primers in the mouse β-globin locus were represented by vertical bars and horizontal arrows respectively. (**B**) The black shading represents the anchor fragment for β-major-globin gene in PCR. Relative cross-linking frequency with fragments for the LCR HSs was presented at unindicted stage and three stages after transcriptional induction. (**C**) The black shading represents the anchor fragment and the grey shadings are fragments generated by *Hin*dIII digestion. Relative cross-linking frequency with fragments between the LCR HSs was additionally presented at uninduced and 72 h induced stages. Relative cross-linking frequency was determined by quantitatively comparing ligated DNA in cross-linked chromatin with control DNA and then by normalizing to the cross-linking frequency in the Ercc3 gene. The results are averages of 5–6 independent experiments ± S.E.M.

Chromatin loop structure of the mouse β-globin locus was analysed by the 3C assay using *Hin*dIII enzyme that separates the LCR HSs into different fragments (Figure 2A). In the assay, cross-linking frequency between the β-major-globin gene and LCR HSs was sequentially increased after transcriptional induction (Figure 2B). The strongest increase was observed at 24 h in HS3, HS2 and HS1. The further increases were observed at 48 and 72 h. Two fragments containing HS4, HS5 and HS6 did not show any significant change in cross-linking frequency with the β-major-globin gene. This is likely to be because the HS5 and HS6 are insulators, not enhancers. Thus, these results indicate that chromatin looping of the β-major-globin gene with LCR HSs precedes the transcriptional activation of gene.

Close positioning of the LCR HSs with actively transcribed globin genes was showed in many studies. However it is not clear whether the intervening regions between the HSs are also closely positioned with the target genes. We analysed cross-linking frequency of the intervening regions among the HS3, HS2 and HS1 with the β-major-globin gene. The frequency was relatively low and not increased after transcriptional induction (Figure 2C). These results indicate that only HSs are closely positioned with the target gene by looping out intervening regions between them.

### eRNAs on the LCR HSs are transcribed before the transcriptional activation of gene in the mouse β-globin locus

**Figure 3:**
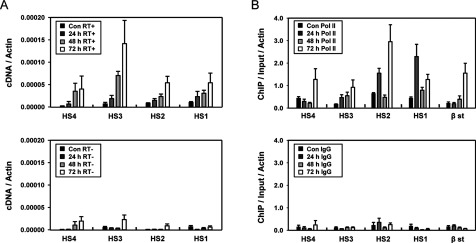
Transcription on the mouse β-globin LCR HSs in HMBA-treated MEL cells (**A**) RT-PCR was performed in MEL cells at the four stages before and after transcriptional induction with or without reverse transcriptase (RT^+^ or RT^−^) and analysed as described in Figure 1(B). (**B**) ChIP was performed with antibodies specific to RNA polymerase II over a time course. Relative intensity was determined by quantitatively comparing immunoprecipitated DNA with input for the indicated amplicons and normalizing to the intensity at the actin gene. Normal rabbit IgG (IgG) served as experimental control. The results are average of 3–4 independent experiments ± S.E.M.

To investigate the kinetics of eRNA transcription during transcriptional induction, transcripts from the mouse β-globin LCR were analysed in HMBA-treated MEL cells. RNA was extracted before and after transcriptional induction at every 24 h and cDNA was generated using random hexamers. Quantitative PCR showed that transcripts from the LCR HSs are sequentially increased after transcriptional induction (Figure 3A); although the amount of transcripts was lower than the mRNA amount of the βmajor globin in uninduced MEL cells (Figure 1B). The increase in eRNAs was detected at 24 h after transcriptional induction, even though the transcription of the β-major-globin gene was not increased at this stage. Thus these results reveal that transcription on the LCR HSs occurs by transcriptional induction and precedes the transcriptional activation of gene in the mouse β-globin locus.

To support the increases in eRNAs on the β-globin LCR, we examined the association of RNA polymerase II at the LCR HSs. ChIP was performed with antibodies to RNA polymerase II in HMBA-induced MEL cells. RNA polymerase II was detected at very low levels in the LCR HSs before transcriptional induction, but the levels were strongly increased at 24 h after the induction in the LCR HS3, HS2 and HS1, even not in HS4 (Figure 3B). High levels of RNA polymerase II were observed at 48 or 72 h. At the transcription start site of the β-major-globin gene, RNA polymerase II was increased at 48 h and further increased at 72 h. The association pattern of RNA polymerase II over the time course was generally parallel with the transcription pattern of the LCR HSs and globin gene. The level of RNA polymerase II detected by ChIP assay was unexpectedly high in the LCR HSs compared with the amount of transcribed eRNAs.

## DISCUSSION

**Figure 4:**
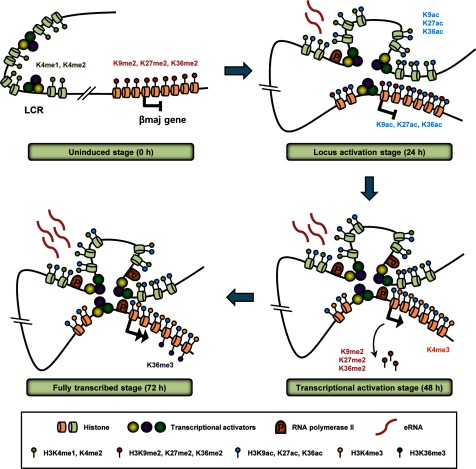
Changes of chromatin structure during transcriptional induction in the mouse β-globin locus The results presented in Figure 1–3 and in our previous study were combined and represented in the mouse β-globin locus [28]. The four stages were named as uninduced, locus activation, transcriptional activation and fully transcribed stages.

The transcriptional activation of gene in eukaryotes accompanies numerous events including transcription factor binding, nucleosome remodelling, histone modifications, chromatin looping and eRNA transcription. The order of these events is one of the most basic and common questions. The present study, together with our previous study [28], shows the dynamics of chromatin structure and coding and non-coding RNA transcription during transcriptional induction over 72 h in the mouse β-globin locus (Figure 4). We have named the four stages as uninduced, locus activation, transcriptional activation and fully transcribed stages. The most notable point is that events on the LCR occur at a locus activation stage that is after transcriptional induction but before transcriptional activation of gene. The events include chromatin looping of the LCR HSs with target gene and transcription of eRNAs. It suggests that these events are pre-requisite for active gene transcription rather than consequence of it.

Precedence of chromatin looping and eRNA transcription to the transcriptional activation of gene implies that they have roles in preparing active gene transcription. Physical interaction of enhancer with promoter through co-activators, such as LDB1 (LIM domain-binding protein 1), appears to promote the recruitment of RNA polymerase II to promoter and/or its elongation into the gene [6,33]. RNA polymerase II recruited to enhancer can be transferred to promoter by looping or tracking [17,34]. Indeed, RNA polymerase II is found in the LCR HSs before the transcriptional activation of gene in the β-globin locus (Figure 4). eRNA transcription might play a role in modifying histones across the enhancer or locus. Acetylation and demethylation at several lysine residues of histone H3 in the LCR HSs are established at 24 h after transcriptional induction in concomitance with eRNA transcription [28]. These modifications could be conferred by histone-modifying enzymes that associate with RNA polymerase II elongating on enhancer. The physical association of histone acetyltransferase or methyltransferase with elongating polymerases has been reported [35,36]. In this case, eRNAs could be by-products of RNA polymerase II elongation for establishing active histone modifications in enhancers.

The concomitant occurrence of the two events, chromatin looping and eRNA transcription, in the β-globin locus suggests functional relationship between them. Genome wide analysis shows that transcription of eRNAs is correlated with chromatin looping of enhancers with target gene promoters [37]. Studies using knockdown of eRNAs indicate that eRNAs, products of transcription, exerts a direct role in chromatin looping. Removal of eRNAs using siRNAs or LNAs (locked nucleic acids) causes a loss in chromatin loops that are formed by oestradiol treatment in human breast cancer cells [38]. eRNAs (non-coding RNA-activating) interact with mediator, multi-subunit co-activator complex bridging transcriptional activators and RNA polymerase II that is required for chromatin looping between the loci transcribing eRNAs and their target genes [39]. However, studies using inhibitors to RNA polymerase II elongation show that chromatin loops are formed independently from polymerase II elongation or eRNA and/or coding RNA production in the gene loci induced by androgen receptor or oestrogen receptor [34,37]. Further studies might be required to elucidate whether eRNAs play a role in chromatin loop formation or the transcription process of eRNAs does it.

## Online data

Supplementary data

## References

[1] Plank J.L., Dean A. (2014). Enhancer function: mechanistic and genome-wide insights come together. Mol. Cell.

[2] Marsman J., Horsfield J.A. (2012). Long distance relationships: enhancer-promoter communication and dynamic gene transcription. Biochim. Biophys. Acta.

[3] Song S.H., Hou C., Dean A. (2007). A positive role for NLI/Ldb1 in long-range β-globin locus control region function. Mol. Cell.

[4] Yun W.J., Kim Y.W., Kang Y., Lee J., Dean A., Kim A. (2014). The hematopoietic regulator TAL1 is required for chromatin looping between the β-globin LCR and human γ-globin genes to activate transcription. Nucleic Acids Res..

[5] Vakoc C.R., Letting D.L., Gheldof N., Sawado T., Bender M.A., Groudine M., Weiss M.J., Dekker J., Blobel G.A. (2005). Proximity among distant regulatory elements at the β-globin locus requires GATA-1 and FOG-1. Mol. Cell.

[6] Deng W., Lee J., Wang H., Miller J., Reik A., Gregory P.D., Dean A., Blobel G.A. (2012). Controlling long-range genomic interactions at a native locus by targeted tethering of a looping factor. Cell.

[7] Kim T.K., Hemberg M., Gray J.M., Costa A.M., Bear D.M., Wu J., Harmin D.A., Laptewicz M., Barbara-Haley K., Kuersten S. (2010). Widespread transcription at neuronal activity-regulated enhancers. Nature.

[8] De Santa F., Barozzi I., Mietton F., Ghisletti S., Polletti S., Tusi B.K., Muller H., Ragoussis J., Wei C.L., Natoli G. (2010). A large fraction of extragenic RNA pol II transcription sites overlap enhancers. PLoS Biol..

[9] Ørom U.A., Derrien T., Beringer M., Gumireddy K., Gardini A., Bussotti G., Lai F., Zytnicki M., Notredame C., Huang Q. (2010). Long noncoding RNAs with enhancer-like function in human cells. Cell.

[10] Melo C.A., Drost J., Wijchers P.J., van de Werken H., de Wit E., Oude Vrielink J.A., Elkon R., Melo S.A., Léveillé N., Kalluri R. (2013). eRNAs are required for p53-dependent enhancer activity and gene transcription. Mol. Cell.

[11] Kaikkonen M.U., Spann N.J., Heinz S., Romanoski C.E., Allison K.A., Stender J.D., Chun H.B., Tough D.F., Prinjha R.K., Benner C. (2013). Remodeling of the enhancer landscape during macrophage activation is coupled to enhancer transcription. Mol. Cell.

[12] Mousavi K., Zare H., Dell'orso S., Grontved L., Gutierrez-Cruz G., Derfoul A., Hager G.L., Sartorelli V. (2013). eRNAs promote transcription by establishing chromatin accessibility at defined genomic loci. Mol. Cell.

[13] Lam M.T., Cho H., Lesch H.P., Gosselin D., Heinz S., Tanaka-Oishi Y., Benner C., Kaikkonen M.U., Kim A.S., Kosaka M. (2013). Rev-Erbs repress macrophage gene expression by inhibiting enhancer-directed transcription. Nature.

[14] Palstra R.J., Tolhuis B., Splinter E., Nijmeijer R., Grosveld F., de Laat W. (2003). The β-globin nuclear compartment in development and erythroid differentiation. Nat. Genet..

[15] Tolhuis B., Palstra R.J., Splinter E., Grosveld F., de Laat W. (2002). Looping and interaction between hypersensitive sites in the active β-globin locus. Mol. Cell.

[16] Du M.J., Lv X., Hao D.L., Zhao G.W., Wu X.S., Wu F., Liu D.P., Liang C.C. (2008). MafK/NF-E2 p18 is required for β-globin genes activation by mediating the proximity of LCR and active β-globin genes in MEL cell line. Int. J. Biochem. Cell. Biol..

[17] Johnson K.D., Christensen H.M., Zhao B., Bresnick E.H. (2001). Distinct mechanisms control RNA polymerase II recruitment to a tissue-specific locus control region and a downstream promoter. Mol. Cell.

[18] Kim A., Dean A. (2004). Developmental stage differences in chromatin sub-domains of the β-globin locus. Proc. Natl. Acad. Sci. U.S.A..

[19] Ashe H.L., Monks J., Wijgerde M., Fraser P., Proudfoot N.J. (1997). Intergenic transcription and transinduction of the human β-globin locus. Genes Dev..

[20] Johnson K.D., Grass J.A., Park C., Im H., Choi K., Bresnick E.H. (2003). Highly restricted localization of RNA polymerase II within a locus control region of a tissue-specific chromatin domain. Mol. Cell. Biol..

[21] Kong S., Bohl D., Li C., Tuan D. (1997). Transcription of the HS2 enhancer toward a cis-linked gene is independent of the orientation, position, and distance of the enhancer relative to the gene. Mol. Cell. Biol..

[22] Plant K.E., Routledge S.J., Proudfoot N.J. (2001). Intergenic transcription in the human β-globin gene cluster. Mol. Cell. Biol..

[23] Ling J., Baibakov B., Pi W., Emerson B.M., Tuan D. (2005). The HS2 enhancer of the β-globin locus control region initiates synthesis of non-coding, polyadenylated RNAs independent of a cis-linked globin promoter. J. Mol. Biol..

[24] Kim A., Zhao H., Ifrim I., Dean A. (2007). β-globin intergenic transcription and histone acetylation dependent on an enhancer. Mol. Cell. Biol..

[25] Forsberg E.C., Downs K.M., Christensen H.M., Im H., Nuzzi P.A., Bresnick E.H. (2000). Developmentally dynamic histone acetylation pattern of a tissue-specific chromatin domain. Proc. Natl. Acad. Sci. U.S.A..

[26] Zhou Z., Li X., Deng C., Ney P.A., Huang S., Bungert J. (2010). USF and NF-E2 cooperate to regulate the recruitment and activity of RNA polymerase II in the β-globin gene locus. J. Biol. Chem..

[27] Fromm G., de Vries C., Byron R., Fields J., Fiering S., Groudine M., Bender M.A., Palis J., Bulger M. (2009). Histone hyperacetylation within the β-globin locus is context-dependent and precedes high-level gene expression. Blood.

[28] Kim K., Kim A. (2010). Sequential changes in chromatin structure during transcriptional activation in the β globin LCR and its target gene. Int. J. Biochem. Cell Biol..

[29] Heo H.S., Kim J.H., Lee Y.J., Kim S.H., Cho Y.S., Kim C.G. (2005). Microarray profiling of genes differentially expressed during erythroid differentiation of murine erythroleukemia cells. Mol. Cells.

[30] Kim Y.W., Kim S., Kim C.G., Kim A. (2011). The distinctive roles of erythroid specific activator GATA-1 and NF-E2 in transcription of the human fetal γ-globin genes. Nucleic Acids Res..

[31] Hagège H., Klous P., Braem C., Splinter E., Dekker J., Cathala G., de Laat W., Forné T. (2007). Quantitative analysis of chromosome conformation capture assays (3C-qPCR). Nat. Protoc..

[32] Cho Y., Song S.H., Lee J.J., Choi N., Kim C.G., Dean A., Kim A. (2008). The role of transcriptional activator GATA-1 at human β-globin HS2. Nucleic Acids Res..

[33] Krivega I., Dale R.K., Dean A. (2014). Role of LDB1 in the transition from chromatin looping to transcription activation. Genes Dev..

[34] Wang Q., Carroll J.S., Brown M. (2005). Spatial and temporal recruitment of androgen receptor and its coactivators involves chromosomal looping and polymerase tracking. Mol. Cell.

[35] Wittschieben B.O., Otero G., de Bizemont T., Fellows J., Erdjument-Bromage H., Ohba R., Li Y., Allis C.D., Tempst P., Svejstrup J.Q. (1999). A novel histone acetyltransferase is an integral subunit of elongating RNA polymerase II holoenzyme. Mol. Cell.

[36] Li J., Moazed D., Gygi S.P. (2002). Association of the histone methyltransferase Set2 with RNA polymerase II plays a role in transcription elongation. J. Biol. Chem..

[37] Hah N., Murakami S., Nagari A., Danko C.G., Kraus W.L. (2013). Enhancer transcripts mark active estrogen receptor binding sites. Genome Res..

[38] Li W., Notani D., Ma Q., Tanasa B., Nunez E., Chen A.Y., Merkurjev D., Zhang J., Ohgi K., Song X. (2013). Functional roles of enhancer RNAs for oestrogen-dependent transcriptional activation. Nature.

[39] Lai F., Orom U.A., Cesaroni M., Beringer M., Taatjes D.J., Blobel G.A., Shiekhattar R. (2013). Activating RNAs associate with mediator to enhance chromatin architecture and transcription. Nature.

