# Supplementary material for: Chromatin looping and eRNA transcription precede the transcriptional activation of gene in the β-globin locus
Source: Biosci Rep. 2015 Mar 18;35(2):e00179. doi: 10.1042/BSR20140126 (PMC4370096; doi:10.1042/BSR20140126)
Supplement: Supplementary data [file bsr035e179ntsadd.pdf]

Supplementary Table 1. Primers of 3C

Amplicon	Primers
HS out	5′ TTG CAT GAT AAA CGT GAA TGA A 3′
HS6	5′ CTG ACT CAG TTC CTT GGA AGA 3′
HS5/4	5′ CGG AAG AAA TCA CTC CTG CT 3′
HS3	5′ AGC CTT CCT CTC TGC CTT GT 3′
HS3 3′	5′ AAG GGC AGG GAC TGC TTA TC 3′
HS2	5′ GCA AGG CTG TTA TCC CTT T 3′
HS2 3′	5′ TGG CTT GTG CTG TAA GAT CG 3′
HS1	5′ AAA GCC ATT CTG AAA GTA AG 3′
εy 3′	5′ TCC CAG AGT TTC TCC TCC AA 3′
β maj	5′ CTC AAT CAA CTA CTG AAT TGG TGT 3′
